# PSMD2 promotes the progression of bladder cancer and is correlated with immune infiltration

**DOI:** 10.3389/fonc.2022.1058506

**Published:** 2022-11-23

**Authors:** Song Wang, He Wang, Shaoxing Zhu, Zongping Wang

**Affiliations:** ^1^ Department of Urology, Zhejiang Cancer Hospital, Institute of Basic Medicine and Cancer (IBMC), Chinese Academy of Sciences, Hangzhou, China; ^2^ The Second Clinical Medical College, Zhejiang Chinese Medical University, Hangzhou, China

**Keywords:** PSMD2, bladder cancer, progression, immune infiltration, treatment

## Abstract

**Introduction:**

PSMD2 plays an oncogenic role in multiple human malignancies, while it is still unclear that the potential roles and underlying mechanisms of PSMD2 in BCa.

**Methods:**

The RNA-seq from TCGA and GTEx database was utilized to preliminarily analyze the expression of PSMD2 in BCa tissues, qRT-PCR was adopted to verify the PSMD2 expression in BCa cell lines. Cox regression analyses were applied to assess the prognostic values of PSMD2 in BCa. GSEA analysis was used to explore the underlying mechanisms of PSMD2. *In vitro* assays such as wound healing and colony formation assays were applied to determine the carcinogenesis of PSMD2 in BCa. xCell and ssGSEA algorithms were applied to analyze the associations of PSMD2 with TIME.

**Results:**

The results revealed that in comparison with normal bladder tissues and cell line, PSMD2 was found to be significantly elevated in BCa tissues and cell lines. Elevated expression of PSMD2 can independently predict unfavorable OS for BCa patients. The PSMD2 expression and other clinicopathologic factors were combined to develop a nomogram, which can help to predict OS for BCa patients. GSEA analyses revealed that PSMD2 is correlated with the cell cycle, antigen processing and presentation, JAK-STAT signaling pathway, Toll like receptor signaling pathway, P53 and MAPK signaling pathway. Knockdown of PSMD2 could remarkably inhibit the wound healing and colony formation efficiency of BCa cells. xCell analysis revealed that overexpressed PSMD2 is positively related to the Th2 cells infiltrates and expression levels of immune escape markers, and negatively associated with the infiltrating levels of NK T cell and CD8+ T cell.

**Discussion:**

In conclusion, overexpressed PSMD2 is tightly linked to the immune infiltrates and promotes the progression of BCa.

## Introduction

Bladder cancer (BCa) is one of the most common malignancies and causes nearly 573,000 new cases and 213,000 deaths annually (1). Although the diagnosis and treatment have improved in recent years, BCa is still a serious threat to the lives of patients and affects their life quality, especially for elder muscle invasive bladder cancer (MIBC) patients. The intracavity Bacillus Calmette-guerin (BCG) has long been considered as an effective treatment for BCa (2), which was reported to activate the immune system and induce inflammation to exert anti-tumor effects (3), while the efficiency of the immune system declines with age. Recently, studies focus on immunotherapy of cancer have developed rapidly, especially for the treatment of BCa, and inhibitors of immunological markers have shown powerful therapeutic effects in BCa (4, 5). In the IMvigor010 study, MIBC patients treated with atezolizumab were reported to possess longer disease-free survival time in comparison with observation group (19.4 vs 16.6 months) (6). Meanwhile, a multicenter single arm phase 2 study showed that the combination of ipilimumab could improve therapeutic response by 22% and prolong the survival of BCa patients (7). However, there are still many patients with advanced BCa who are not sensitive to immunotherapy and lost their last chance of treatment (8). Therefore, it is vital important to find prognostic and immunological targets to prolong survival time for advanced BCa patients.

PSMD2 belongs to the proteasome 26S subunit. Emerging evidence shows PSMD2 is overexpressed in human cancers, including lung cancer (9), gastric cancer (10), breast cancer (11), which implies its vital roles in carcinogenesis. Additionally, more and more studies report that PSMD2 is tightly linked with immunosuppression in multiple cancer types (12, 13). However, it is still unclear that the potential roles and underlying mechanisms of PSMD2 in BCa.

In this study, we demonstrated that the upregulated PSMD2 expression in BCa by analyzing the data from databases and verified the elevated expression level of PSMD2 in BCa cell lines. Overexpressed PSMD2 can independently predict unfavorable overall survival for BCa patients. *In vitro* assays such as wound healing and colony formation assays were also utilized to determine the carcinogenic functions of PSMD2 in BCa cells. Subsequently, xCell and ssGSEA algorithms were applied to explore correlations between PSMD2 and TIME of BCa. This study showed that PSMD2 promotes BCa progression and shapes the TIME, which might be a potential target for BCa treatment.

## Materials and methods

### Data analysis

The data of more than 30 types of cancer patients were downloaded from the GTEx database (https://gtexportal.org/) and TCGA database (https://portal.gdc.cancer.gov/). The GSE13507 from the GEO database (https://www.ncbi.nlm.nih.gov/geo/) and the Kaplan-Meier plotter database (http://kmplot.com/analysis/) was adopted to verify the prognostic significance of PSMD2 in BCa. Differentially genes of PSMD2 high and low BCa samples were performed by using the “Limma” package, and setting the |log2(FC)|>1 and p. adj<0.05 as the threshold.

### Cell culture and transfection

Human BCa cell lines (UC3, T24, J82) and normal bladder cell line (SV) were obtained from the Cell Bank of the Chinese Academy of Sciences (Shanghai, China) and cultivated as previously reported (14), the minimum essential medium (MEM) (Corning) was used to culture UC3 and J82 cell lines, and RPMI 1640 medium (Corning) was used to culture T24 and SV cell lines. These cells were cultured with medium and 10% sterilized fetal bovine serum (FBS, BI) at 5% CO2 and 37°C in a humidified atmosphere. Transfection cell experiments were performed by using the Polyplus transfection^®^ reagent (Proteintech), the siRNAs were synthesized in TsingKe (Hangzhou, China) and the sequences were listed in (**Table S1**).

### qRT-PCR analysis

Total RNA was extracted by using TRIzol agentia (Takara), the PrimeScript RT reagent Kit (Takara) was used to perform reverse transcription, the SYBR Premix Ex Taq (Takara) was used to perform PCR in thermocycler, and the relative expression levels of PSMD2 were detected *via* qRT-PCR method as previous described (15), GAPDH was selected as the endogenous reference. All primers were included in **Table S1**.

### Wound healing and colony formation assay

After the transfection procedure for 48 h, transfected cells were counted and conducted wound healing and colony formation assay, for colony formation assay, 500 treated cells per well were counted and cultured in 6-well plates, after cultured for 7-14 days, cells were fixed with methanol for 10 minutes and then stained with 0.3% crystal violet for 10 minutes, 6-well plates were washed and counted. For wound healing assay, the treated cells grew to 100% confluence in 6-well plates, and sterile tip was used to make a scratch on the plate to generate wounds. Cells were then maintained in serum-free medium for 24h and visualized using a phase-contrast microscopy, the ranges of wound healing were used to assess the ability of wound healing.

### Cox analyses and development of nomogram

Cox analysis was adopted to explore the prognosis of PSMD2 in 33 types of tumor, including BCa. The forest plots were applied to exhibit the HR and P value of each character. With the help of the “rms” package, the nomogram was constructed to assess the OS of BCa patients through integrating PSMD2 expression and other clinicopathological factors, and the goal of a nomogram is to help predict individual BCa patient’s 1-year, 3-year and 5-year survival probability.

### GSEA

In order to investigate the possible roles and underlying mechanisms of PSMD2 in BCa, the “Cluster Profiler” was used to perform the GO and GSEA analyses (16) to find the GO and KEGG pathways of PSMD2 in BCa. For GSEA analysis, the permutation tests were performed 1,000 times, and p-values were adjusted for multiple testing by performing the Benjamini–Hochberg procedure, p < 0.05 and FDR < 0.05 was a significantly enriched pathway.

### Tumor Immune Microenvironment (TIME) analysis

TIME is an important regulatory factor of tumor development, and tumor immune infiltrates are main component of TIME. To carry out a calculable evaluation of tumor immune infiltrates, the connections between PSMD2 and its top 6 coexpressed genes and TIME in BCa were firstly assessed *via* utilizing the ssGSEA algorithm (17). Then the BCa samples were separated into two PSMD2 groups based on median PSMD2 expression in TCGA-BLCA cohort, the xCell algorithm (18) was applied to evaluate the TIME component of this two PSMD2 groups *via* using the “immunedeconv” package and visualized by the “pheatmap” package. Meanwhile, the tumor immune dysfunction and exclusion (TIDE) (19) algorithm was adopted to assess the therapeutic effect of immunotherapy. TIDE uses a panel of gene expression signatures to evaluate the mechanisms of tumor immune escape, including dysfunction of tumor-infiltrating cytotoxic T lymphocytes (CTLS) and CTL rejection by immunosuppressive factors. The higher the TIDE score, the lower the patients’ sensitivity to immune checkpoint blocking therapy, and shorter survival after ICB treatment. Additionally, the correlation between PSMD2 expression with immune signature in pan-cancer (20) was also evaluated *via* utilizing data compiled by TCGA cohorts.

### Statistical analysis

GraphPad Prism 8.0 and (version 3.6.3) R were used to perform the statistics. Wilcox test was applied to conduct comparisons among groups. Pearson analyses were utilized to evaluate associations of PSMD2 with others in BCa. P < 0.05 represented meaningful statistics.

## Results

### PSMD2 is significantly upregulated in BCa

**Figure 1:**
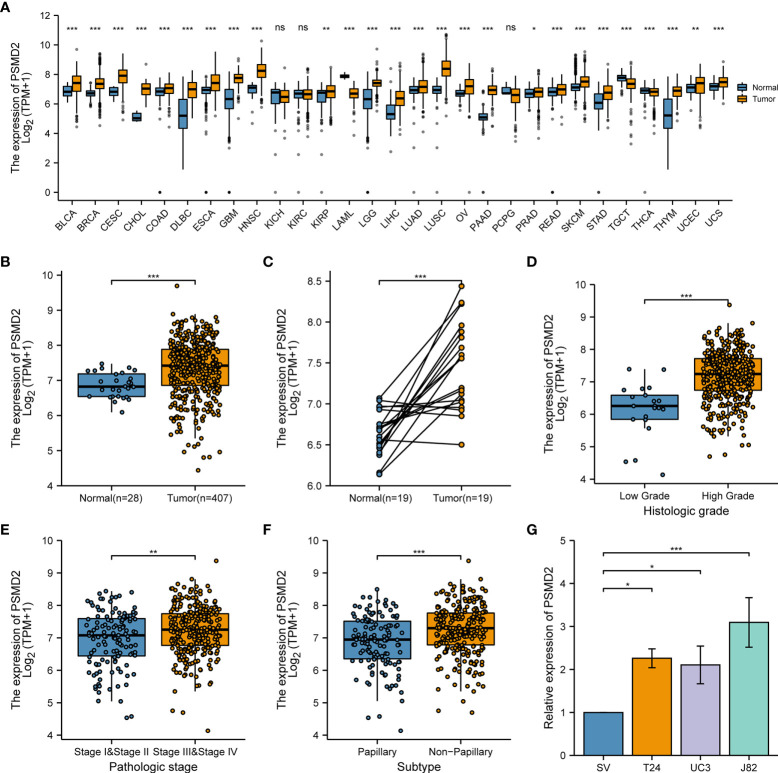
PSMD2 is significantly upregulated in BCa tissues and cell lines. **(A)** The relative expression of PSMD2 in most types of cancer tissues compared with normal tissues in TCGA and GTEx database. The orange color represents tumor tissues and the blue color represents normal tissues. In comparison with normal bladder tissues, the relative expression of PSMD2 in **(B)** non-paired BCa tissues and **(C)** paired BCa tissues. The relative expression of PSMD2 in different **(D)** tumor histologic grade and **(E)** pathologic stage BCa tissues. **(F)** The relative expression of PSMD2 in papillary BCa tissues and non-papillary BCa tissues. **(G)** qRT-PCR analysis reveals the relative expression level of PSMD2 in BCa cell lines (T24, UC3, J82) compared with normal bladder cell line (SV). ns p>0.05, *p < 0.05; **p < 0.01, ***p < 0.001.

Through analyzing the transcriptional data of the GTEx and TCGA database, the expression values of PSMD2 were firstly investigated in multiple cancers involving over 10000 cancer and normal tissue samples. As shown in **Figure 1A**, PSMD2 was remarkably overexpressed in most cancer types, including bladder cancer (**Figure 1B**). Meanwhile, PSMD2 was also found to be upregulated in 19 paired BCa and normal bladder tissue samples in the TCGA database (**Figure 1C**). Elevated PSMD2 expression was correlated with advanced tumor grade (**Figure 1D**) and pathologic stage (**Figure 1E**). Moreover, compared with papillary BCa, PSMD2 was significantly upregulated in non-papillary BCa (**Figure 1F**). To verify the expression level of PSMD2 *in vitro*, RT-qPCR was applied to detect relative PSMD2 transcriptional expression in BCa cells and normal bladder cell line, and the results revealed that PSMD2 is significantly upregulated in BCa cells (**Figure 1G**). These findings demonstrated that PSMD2 is overexpressed in BCa.

### Upregulation of PSMD2 predicts poor survival and development of nomogram model

**Figure 2:**
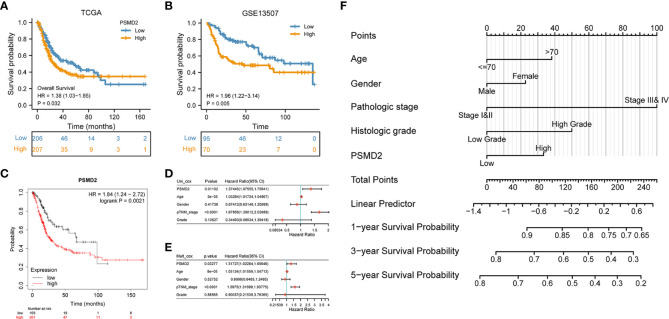
Upregulation of PSMD2 is an independent prognostic factor for BCa. Different expression level of PSMD2 indicates distinct 5-year survival for BCa patients in **(A)** TCGA-BLCA, **(B)** GSE13507 dataset, and **(C)** KM plotter database. **(D)** Univariate and **(E)** multivariate analysis show that upregulation of PSMD2 is an independent prognostic factor for BCa. **(F)** The expression level of PSMD2 is combined with other clinicopathologic factors to develop a nomogram to predict the OS of BCa patients. According to the expression level of PSMD2 in individual patient, and the clinical factors (such as patient’s age, gender, pathologic stage and histologic grade), doctor can calculate the total points of every clinical features, which would assist in evaluating the 1-year, 3-year and 5-year survival probability of BCa patients.

The prognostic values of PSMD2 in BCa were subsequently investigated in multiple datasets. Firstly, the TCGA-BLCA cohort (P=0.032) and GSE13507 dataset (P=0.005) both showed that BCa patients with higher expression level of PSMD2 showed more worse 5-year survial compared with patients with lower PSMD2 expression (**Figures 2A, B**), these were consistent with the Kaplan–Meier plotter database (P=0.0021; **Figure 2C**). Additionally, the COX analysis was also adopted to investigate the prognosis of PSMD2 expression and other clinical parameters in BCa. As plotted in **Figure 2D**, the univariate analysis showed that the TNM stage (p < 0.0001), PSMD2 expression (P=0.01102) and age (p=3e-05) are related to the OS of BCa patients (**Figure 2D**), and multivariate analyses also demonstrated that the TNM stage, age and PSMD2 expression (p<0.05) are independent factors for predicting the OS of BCa patients (**Figure 2E**). Furthermore, a nomogram model involving PSMD2 expression and other clinical factors was also been constructed (**Figure 2F**), according to the expression level of PSMD2 in individual patient, and the clinical factors (such as patient’s age, gender, pathologic stage and histologic grade), doctor can calculate the total points of every clinical features, which would assist in evaluating the 1-year, 3-year and 5-year survival probability of BCa patients.

### Differential and GSEA analysis of PSMD2

**Figure 3:**
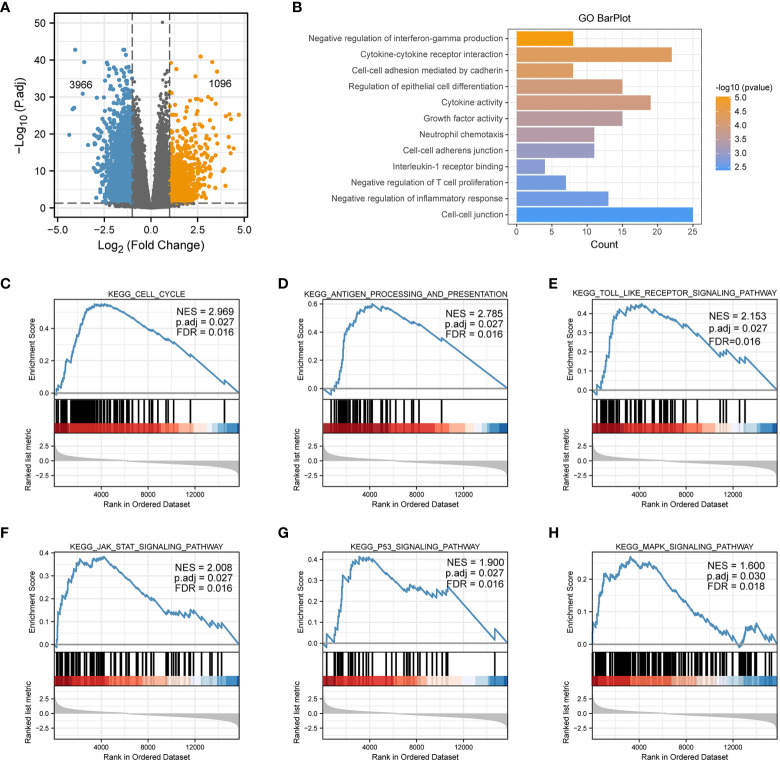
The GO and GSEA analysis of elevated expression of PSMD2 in BCa. **(A)** A volcano plot shows that differentially expressed genes in PSMD2 high and low BCa samples, threshold: |log2(FC)|>1, p. adj<0.05. **(B)** The GO analysis of elevated expression of PSMD2 in BCa. The KEGG signaling pathways of GSEA analysis of **(C)** Cell cycle, **(D)** Antigen processing and presentation, **(E)** Toll like receptor signaling pathway, **(F)** JAK_STAT signaling pathway, **(G)** P53 signaling pathway, **(H)** MAPK signaling pathway.

To explore possible roles and underlying functions of PSMD2 in BCa, differential analyses between PSMD2 high and low BCa samples was firstly performed, as plotted in **Figure 3A**, when setting the |log2(FC)|>1 and p. adj<0.05 as the threshold, 1096 genes were noted to be upregulated, and 3968 genes were downregulated. The Gene Ontology (GO) analysis revealed that the highly expressed PSMD2 related pathways are Negative regulation of interferon-gamma production, Cytokine-cytokine receptor interaction, Negative regulation of T cell proliferation, Cell-cell adhesion mediated by cadherin and et al. (**Figures 3B**). Moreover, GSEA analysis showed that the Cell cycle (NES=2.969), Toll-like receptor (NES=2.153), Antigen processing and presentation (NES=2.785), JAK-STAT (NES=2.008), P53 (NES=1.9) and MAPK signaling pathway (NES=1.6) (**Figures 3C–H**) are significantly centralized in overexpressed PSMD2 BCa samples. These findings revealed that the cell cycle and regulation of tumor immune infiltration pathways are tightly linked with the abnormally elevated PSMD2 in BCa.

### Knockdown of PSMD2 inhibits the progression of BCa cells

**Figure 4:**
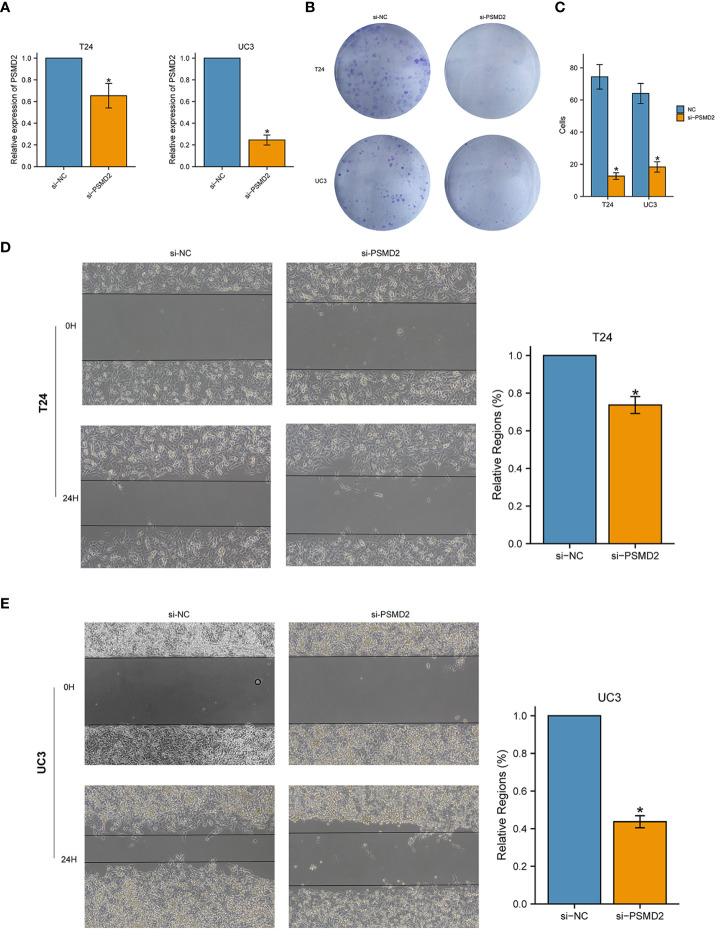
Silencing the expression of PSMD2 significantly inhibits the tumorigenicity of BCa cells. The relative expression level of PSMD2 in **(A)** T24 cell line and **(B)** UC3 cell line when treated with si-PSMD2 and si-NC. **(C)** Knockdown of PSMD2 expression weaken the colony formation ability of BCa cells. Knockdown of PSMD2 significantly abate the wound-healing efficiency of **(D)** T24 and **(E)** UC3 cell. *p < 0.05. The orange color represents si-PSMD2 treated group, the blue color represents si-NC treated group.

Considering the elevated expression of PSMD2 may contribute to carcinogenic effect, *in vitro* assays were subsequently performed to verify the effects of PSMD2 in BCa. Firstly, BCa cells UC3 and T24 were transfected with PSMD2 small interference RNA, as plotted in **Figures 4A, B**, the expression level of PSMD2 was remarkably decreased compared to negative control T24 and UC3 cells. Then the si-NC and si-PSMD2 treated T24, UC3 cells were adopted to perform functional assays, including wound healing and colony formation assay. The results demonstrated that the silencing of PSMD2 remarkably weaken the colony formation efficiency (**Figure 4C**) and migration abilities (**Figures 4D, E**) of BCa cells. These findings revealed that the upregulated PSMD2 may be participate the progression of BCa.

### Tight links between PSMD2 and tumor immune infiltrates in BCa

**Figure 5:**
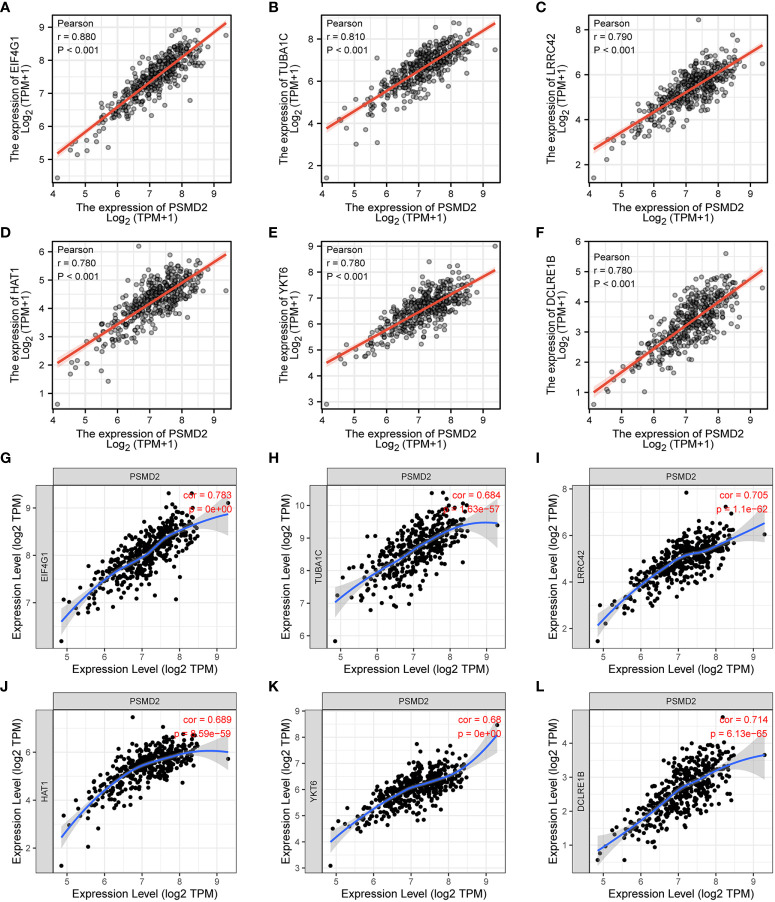
The co-expressed genes of PSMD2 in BCa. The expression correlations of **(A–F)** EIF4G1 (r=0.880, p<0.001), TUBA1C (r=0.810, p<0.001), LRRC42 (r=0.790, p<0.001), HAT1 (r=0.780, p<0.001), YKT6 (r=0.780, p<0.001) and DCLRE1B (r=0.780, p<0.001) with PSMD2 in TCGA-BLCA cohort. **(G–L)** The expression associations of PSMD2 with the EIF4G1 (cor=0.783, p=0), TUBA1C (cor=0.684, p=1.63e-57), LRRC42 (cor=0.705, p=1.1e-62), HAT1 (cor=0.689, p=8.59e-59), YKT6 (cor=0.68, p=0), DCLRE1B (cor=0.714, p=6.13e-65) in BCa in TIMER database.

**Figure 6:**
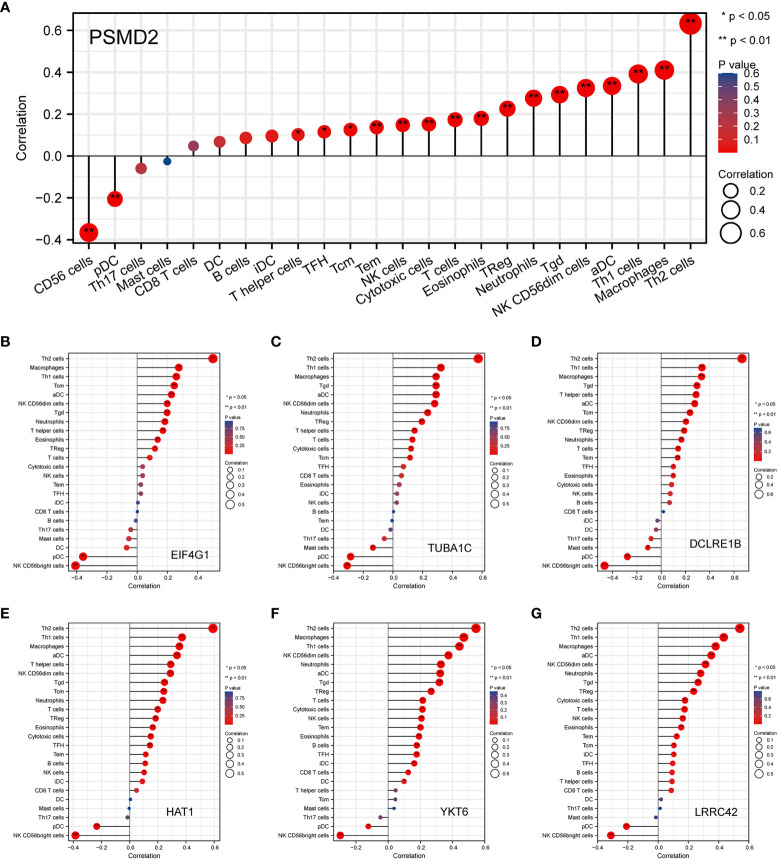
The correlations of PSMD2 and its co-expressed genes with the immune infiltration cells in BCa. The associations of **(A)** PSMD2, **(B)** EIF4G1, **(C)** TUBA1C, **(D)** LRRC42, **(E)** HAT1, **(F)** YKT6, **(G)** DCLRE1B with tumor immune infiltration cells in BCa. *p < 0.05; **p < 0.01.

Given the GO and GSEA analysis results of overexpressed PSMD2 in BCa, the functions of PSMD2 in TIME were further explored. Firstly, the top 6 positively co-expressed genes of PSMD2 were identified by using Pearson correlation analysis, the results showed that the EIF4G1 (r=0.880, p<0.001), tubulin alpha 1c (TUBA1C) (r=0.810, p<0.001), leucine rich repeat containing 42 (LRRC42) (r=0.790, p<0.001), histone acetyltransferase 1 (HAT1) (r=0.780, p<0.001), YKT6 (r=0.780, p<0.001), DNA cross-link repair 1B (DCLRE1B) (r=0.780, p<0.001) are the top 6 co-expressed genes of PSMD2 in TCGA-BLCA cohort (**Figures 5A–F**). The correlations of these 6 genes with PSMD2 in BCa were verified in TIMER database, which also indicated strong associations between PSMD2 and these 6 genes (p<0.05; **Figures 5G–L**). Subsequently, the ssGSEA algorithm was utilized to assess the associations of PSMD2 with its top 6 coexpressed genes and TIME of BCa. The results demonstrated that there are tight links between PSMD2 and 6 coexpressed genes and TIME in BCa, especially the Th2 cells (P<0.01, **Figures 6A–G**). These results implied PSMD2 might be involved in the regulation of TIME in BCa.

**Figure 7:**
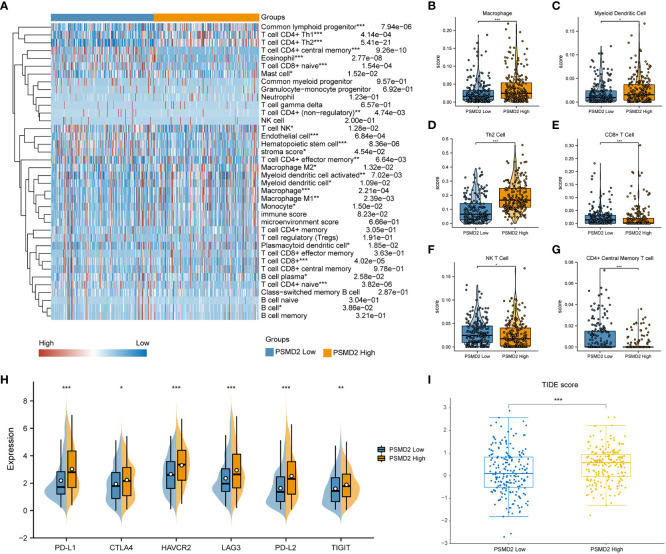
Distinct tumor immune microenvironment in differential PSMD2 expression levels of BCa tissues. **(A)** A heat map visualized distinct infiltrating levels of immune cells in PSMD2 high and low expression BCa samples. The associations of PSMD2 expression with the infiltrating levels of **(B)** Macrophage, **(C)** Myeloid Dendritic cell, **(D)** Th2 cell, **(E)** CD8+ T cell, **(F)** NK T cell, **(G)** CD4+ Central Memory T cell. **(H)** The correlations of PSMD2 expression with the expression levels of immune escape markers (PD-L1, CTLA4, LAG3, HAVCR2, PD-L2, TIGIT) in BCa. **(I)** Tumor Immune Dysfunction and Exclusion (TIDE) analysis reveals distinct TIDE score between different PSMD2 expression BCa tissues. *p < 0.05; **p < 0.01, ***p < 0.001.

Subsequently, the BCa samples were separated into two PSMD2 groups based on its median transcriptional level, namely PSMD2 low and PSMD2 high group, and the xCell algorithm was applied to assess the TIME component of this two PSMD2 group BCa samples. As plotted in the **Figure 7A**, entirely different TIME was noted between the two PSMD2 groups. Specifically, the Macrophage (P<0.001, **Figure 7B**), Myeloid Dendritic cell (P<0.05, **Figure 7C**), and Th2 cell (P<0.001, **Figure 7D**) were significantly enriched in PSMD2 high group, while the CD8+ T cell (P<0.001, **Figure 7E**), NK T cell (P<0.05, **Figure 7F**), and CD4+ Central Memory T cell (P<0.001, **Figure 7G**) were remarkably decreased in PSMD2 high group. Moreover, immune escape markers such as PD-L1, TIGIT, CTLA4, HAVCR2, LAG3, and PD-L2 were remarkably elevated in PSMD2 group (**Figure 7H**). Additionally, the TIDE score was also calculated between two PSMD2 groups, and the results revealed in comparison with PSMD2 low group, the TIDE score is remarkably higher in PSMD2 high group (**Figure 7I**). These results implied that overexpressed PSMD2 might be involved in shaping TIME and related to immune escape in BCa.

### Comprehensive analysis of PSMD2 in pan-cancer

**Figure 8:**
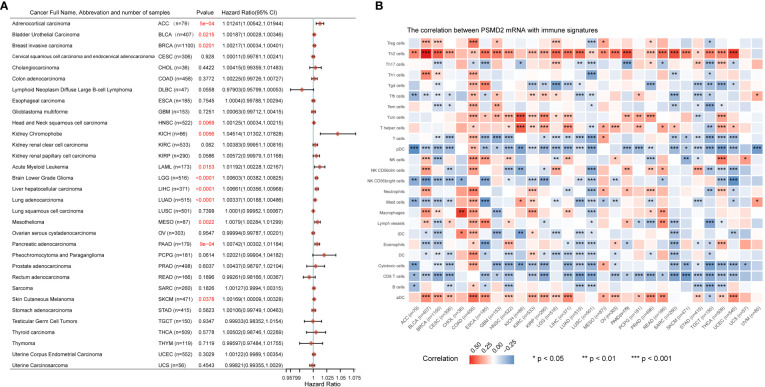
Upregulation of PSMD2 is associated with unfavorable OS and immune infiltration in pan-cancer. **(A)** The associations of PSMD2 expression with overall survival in pan-cancer. The hazard ratio greater than 1 represents its high expression indicating unfavorable OS in patients, while hazard ratio less than 1 represents its high expression suggesting a good OS for patients. The red font indicates p<0.05. **(B)** Pan-cancer analysis shows associations of PSMD2 expression with the infiltrating levels of immune cells in multiple cancer types. Red color represents a positive correlation, blue represents a negative correlation, and darker colors represent stronger correlations. *p < 0.05; **p < 0.01, ***p < 0.001.

Given that the vital roles of PSMD2 in BCa, and the expression levels of PSMD2 were widely elevated in the vast majority of cancers (**Figure 1A**), the roles of PSMD2 were subsequently explored in pan-cancer. The prognostic values of PSMD2 were firstly investigated in more than 30 types of cancer, and the results revealed that the highly expressed PSMD2 predicts unfavorable OS in twelve types of cancer (**Figure 8A**), including the BLCA (Bladder Urothelial Carcinoma), SKCM (Skin Cutaneous Melanoma), HNSC (Head and Neck squamous cell carcinoma), BRCA (Breast invasive carcinoma), LIHC (Liver hepatocellular carcinoma), KICH (Kidney Chromophobe), LAML (Acute Myeloid Leukemia), LGG (Brain Lower Grade Glioma), LUAD (Lung adenocarcinoma), MESO (Mesothelioma), PAAD (Pancreatic adenocarcinoma) and ACC (Adrenocortical carcinoma) (HR>1, P<0.05), while shows no prognostic values in other types of cancer (P>0.05) (**Figure 8A**). Furthermore, the roles of PSMD2 in TIME were also investigated in pan-cancer, as shown in **Figure 8B**, the PSMD2 expression was tightly linked to infiltrates of Th2 cells and is negatively related to CD8+ T cells and cytotoxic cells in the vast majority of cancers (**Figure 8B**). These findings implied that the overexpressed PSMD2 might be implicated in the immune escape in cancer.

## Discussion

Recently, remarkable improvements have been taken place in the diagnosis and treatment of BCa, and novel therapy such as immunotherapy is gradually developed as the standard treatment for advanced BCa patients. However, few patients can acquire sustained clinical benefits from immunotherapies such as the anti-PD1/PD-L1 agents (4). It is vital important to determine novel immunotherapy targets to enhance the clinical outcomes of BCa patients. Here, we demonstrated that PSMD2 is highly expressed in BCa tissues and cell lines in comparison with normal counterparts, upregulation of PSMD2 could serve as an independent predictor for BCa, multiple analyses and cell assays were performed to investigate the carcinogenesis of PSMD2 and its correlations with immune infiltrates.

PSMD2 is considered as a vital important non-ATPase regulatory subunit of the 19S proteasome (9). Previous studies demonstrated that PSMD2 can regulate the cell cycle, apoptosis and proliferation process *via* inducing ubiquitination and degradation of targeted protein (10). In addition to its involvement in proteasome function, this subunit may also be involved in the TNF signaling pathway because it interacts with the tumor necrosis factor type 1 receptor (10). Moreover, PSMD2 was also reported to function as a cancer driver in multiple human malignancies. Tomida et al. identified PSMD2 as an unfavorable prognostic predictor for lung cancer in a genome-wide analysis (21). Matsuyama et al. reported that PSMD2 is tightly linked with clinicopathologic features and might be a potential therapeutic target in lung adenocarcinoma (9). Li et al. demonstrated that PSMD2 promotes breast cancer cell progression by targeting p21 and p27 and inducing their degradations (22). However, the potential functions of PSMD2 in BCa have not been explored. Here, our results revealed that PSMD2 is elevated in BC tissues and cell lines in comparison with normal counterparts, and upregulation of PSMD2 can independently predict dismal OS for BCa patients. Meanwhile, a clinical nomogram was also constructed through integrating PSMD2 expression and other clinicopathological factors in BCa to help make a clinical decision. Moreover, cell assays were performed to verify the carcinogenesis of PSMD2 in human BCa cell lines, the findings showed that PSMD2 depletion can significantly inhibit the wound healing and colony formation ability of BCa cell lines, these results were consistent with previous researches that reported PSMD2 knockdown strongly depress the progression of lung adenocarcinomas, hepatocellular carcinoma and breast cancer (9, 22, 23). Additionally, two recent studies reported that the gene signature based on the PSMD2 expression is tightly linked with prognoses and immune infiltrates in HNSCC and thyroid cancer (12, 13). Therefore, GSEA analysis was applied to investigate the potential functions of PSMD2 in BCa progression, the results implied that the Cell cycle, JAK-STAT signaling pathway, Toll-like receptor signaling pathway, Antigen processing and presentation, P53 and MAPK signaling pathway were remarkably enriched. Toll-like receptor and JAK-STAT signaling pathway were reported to be implicated in regulating tumor microenvironment (24, 25), and previous studies also demonstrated that the JAK-STAT signaling pathway activation acts as an oncogenic role in BCa and other cancers (26–28). these findings implied that PSMD2 might promotes BCa progression and regulates TIME by affecting the JAK-STAT and Toll-like receptor signaling pathway.

To investigate the underlying effects of PSMD2 in BCa, the associations between PSMD2 and its top 6 coexpressed genes and TIME in BCa were initially assessed. The findings demonstrated that there are strong links between PSMD2 and the infiltrating levels of Th2 cells, Th1 cells, activated DC cells and Treg in BCa (**Figure 6**), tumor immunity is a dynamic and balanced process, the dysregulations of PSMD2 expression may lead to both immune system activation and immune suppression. However, in general, PSMD2 may still induce the formation of an immunosuppressive microenvironment, and lead to the elevation of immune suppression checkpoints such as PD-L1 (**Figure 7H**). Interestingly, among the top 6 co-expressed genes of PSMD2, the EIF4G1 and TUBA1C were reported to be related to immune infiltration in lung cancer, and EIF4G1 is significantly associated with the PD-L1 expression (29, 30). Meanwhile, Yang et al. found that YKT6 is related to cell migration and CD8+ T cells infiltration in oral carcinoma (31). Furthermore, the results of xCell algorithm analysis showed distinct TIME between PSMD2 high and low group. The Th2 cell was significantly associated with the expression level of PSMD2, CD8+ T cell and NK T cell were negatively linked to PSMD2 expression. In fact, the NK T cell and CD8+ T cell are considered to be the most important anti-cancer immune cells (20), while the Th2 cell is always act as an immune escape driver in cancer (32). Moreover, the immune checkpoints such as PD-L1 and LAG3 were remarkably enhanced in PSMD2 high group, PD-L1 is recognized as the most important hall marker of immune escape (33) and is correlated with T-cell depletion in multiple cancers, including the BCa (34). These findings implied that PSMD2 might be implicated in immune escape in BCa. Additionally, TIDE analysis demonstrated that the TIDE score is significantly higher in PSMD2 high group in comparison with PSMD2 low group, TIDE score was a model of tumor immune evasion evaluation constructed by Jiang et al. and can assess the therapeutic effect of immune checkpoint blockade with a high accuracy. The higher the TIDE score, the lower the patients’ sensitivity to immune checkpoint blocking therapy (19), these findings demonstrated that BCa patients with high PSMD2 are likely to insensitive to immune checkpoint blocking therapy. Therefore, PSMD2 might be a biomarker for immunotherapy and a therapeutic target for BCa patients.

There are also several defects in this article. Firstly, the analysis was based on the public data from TCGA and GTEx database due to lack of clinical samples in our center. Additionally, although *in vitro* assays and analysis methods were adopted to explore the potential roles of PSMD2 in BCa, the correlations between PSMD2 and TIME should be verified in larger samples clinical trials and basic studies.

## Conclusions

Our study reveals that the abnormally elevated PSMD2 predicts unfavorable survival for BCa patients, and knockdown of PSMD2 could suppress BCa cell progression. Moreover, PSMD2 was found to shape the TIME of BCa and correlated with immune infiltration. Further researches based on clinical trials and experiments should be performed to verify these findings.

## Data availability statement

The datasets presented in this study can be found in online repositories. The names of the repository/repositories and accession number(s) can be found in the article/**Supplementary Material**.

## Author contributions

SW wrote the article. SW and HW performed the data searching. SW conducted the bioinformatics and experimental analysis. ZW and SZ revised the article. All authors approved the final manuscript.

## Funding

The research was funded by the Natural Science Foundation of Zhejiang Province: LQ20H160007.

## Conflict of interest

The authors declare that the research was conducted in the absence of any commercial or financial relationships that could be construed as a potential conflict of interest.

## Publisher’s note

All claims expressed in this article are solely those of the authors and do not necessarily represent those of their affiliated organizations, or those of the publisher, the editors and the reviewers. Any product that may be evaluated in this article, or claim that may be made by its manufacturer, is not guaranteed or endorsed by the publisher.
