# Supplementary material for: PSMD2 promotes the progression of bladder cancer and is correlated with immune infiltration
Source: Front Oncol. 2022 Nov 23;12:1058506. doi: 10.3389/fonc.2022.1058506 (PMC9728585; doi:10.3389/fonc.2022.1058506)
Supplement: Supplementary file 1 [file Table_1.docx]

| **Table S1.** **The Oligonucleotides used in this study.** | | | |
| --- | --- | --- | --- |
| si-RNA | sense（5'-3'） |  | antisense（5'-3'） |
| si-PSMD2-1 | GUGAAGAUGUCCUAACACUTT | | AGUGUUAGGACAUCUUCACTT |
|  |  |  |  |
| si-PSMD2-2 | CCAGUUAGCUCAAUAUCAUTT | | AUGAUAUUGAGCUAACUGGTT |
|  |  |  |  |
| si-PSMD2-3 | GCAUUGAUGCUCAAUGACATT | | UGUCAUUGAGCAUCAAUGCTT |
|  |  | |  |
| Primers | Forward Primer（5'-3'） | | Reverse Primer （5'-3'） |
| PSMD2 | TGCTCGTGGAACGACTAGG | | CAGTTTGCCATAGTGTGGACG |
| GAPDH | CTGGGCTACACTGAGCACC | | AAGTGGTCGTTGAGGGCAATG |
